# Exploration of the Factor Structure of the Burden Experienced by Individuals Providing End-of-Life Care at Home

**DOI:** 10.1155/2018/1659040

**Published:** 2018-07-22

**Authors:** Chizuru Nagata, Hironori Yada, Junko Inagaki

**Affiliations:** ^1^Division of Community/Gerontological Nursing, Yamaguchi University Graduate School of Medicine, 1-1-1 Minami-kogushi, Ube Yamaguchi 755-8505, Japan; ^2^Department of Clinical Nursing, Yamaguchi University Graduate School of Medicine, 1-1-1 Minami-kogushi, Ube Yamaguchi 755-8505, Japan; ^3^Faculty of Nursing, Yamanashi Prefectural University, Kofu City, 1-6-1 Ikeda, Kofu Yamanashi 400-0062, Japan

## Abstract

In Japan, the number of elderly people who require long-term care is increasing as a result of the country's aging population. Consequently, the burden experienced by caregivers who provide end-of-life care at home has become a social problem. This study aimed to confirm the factor structure of such caregiver burden by analyzing the Japanese version of the Zarit Caregiver Burden Interview (J-ZBI). The J-ZBI was administered to 389 caregivers providing end-of-life care, and 247 answers were analyzed, with exploratory factor analysis performed on the results. Consequently, a four-factor structure emerged (sacrificing life, personal strain, severe anxiety, and captivity); these four factors, constituting 15 items, were cumulatively named “J-ZBI_15.” In regard to reliability, Cronbach's *α* coefficient for each factor was high; in terms of validity, a confirmatory factor analysis was conducted to examine the four-factor structure, and the goodness of model fit was determined to be satisfactory. Further, the convergent validity was also high. The care burden experienced by those providing end-of-life care at home differs from the burden of caregivers of individuals with other diseases, such as Alzheimer's. For assessing the burden felt by this population, the 15-item four-factor ZBI model is more appropriate than the single-factor 22-item ZBI, and we also determined that J-ZBI_8 is unsuitable for this task. Thus, measurement of family caregivers' burden in regard to providing end-of-life care at home should be performed using the 15-item four-factor J-ZBI model.

## 1. Introduction

The updated Japanese average lifespan in 2017 recorded an increase, where it was 80.75 years for men, and 86.99 years for women [1]. The population of elderly people aged 65 years or older is approximately 34 million, and aging rate is 27.3%; it is predicted that the population of elderly people aged 65 years or older will be about 37 million in 2025, and that the aging rate will grow to 33.3% in 2036, thereby indicating that one of three Japanese individuals will be elderly [2].

The number of elderly people who require long-term care is increasing; it has risen from 3.9 million in 2003 to approximately 6 million in 2014, as a result of the country's aging population and, with the recent introduction of a policy promoting home medical care, it can be predicted that the number of family caregivers will continue to increase [3]. In 2000, a new care service became available, with the introduction of the Care Insurance System; this system aimed to organize home based care without family caregivers and attempted to provide the necessary care services for the persons aged 40 years and over. However, this service does not cater to home-medical-care needs, and family members must consequently independently bear the burden of caring for elderly family members [4]. There are many studies and reports about care burden, and the Care Insurance System is improving in Japan [5–7]. If for the person who needs intensive nursing care time of care is longer than slight disabled person at home, then family care givers must provide heavy care for person with severe impairment in all day [2]. The issue of home based care causes economic and social problems including abuse or murder by family caregivers, and turnover of them. Therefore, measuring the care burden of family caregivers is essential for preventing deterioration in caregivers' health and for guarding against elderly abuse, and also for developing measures for mitigating the care burden in question. Home care is already regarded as one of the most stressful occupations in Japan and globally as well. It is a significant problem for industrial hygiene.

The Zarit Caregiver Burden Interview (ZBI) was developed by Zarit, Reever, and Bach-Peterson [8]. ZBI originated as a 29-item questionnaire, and later a revised version consisting of 22 items was created [9]. The 22-item ZBI was designed to measure the burden experienced by caregivers of patients with dementia [10–12], but this scale has also been used in various other settings and for other diseases [13]. The 22-item ZBI was translated into Japanese (J-ZBI) [14], and this Japanese version has been used in a number of recent care-burden studies [15–19]; further, it is particularly useful for the evaluation and comparison of care burdens experienced by home caregivers. Additionally, a short Japanese version of the J-ZBI was created (J-ZBI_8), and the reliability and validity of this scale has been confirmed by primary caregivers of older people (n = 735) [20].

These two scales, J-ZBI and J-ZBI_8, have been studied by Arai and Zarit [21] and by Schreiner, Morimoto, Arai, and Zarit [22] in order to calculate the threshold values for scores relating to depressive symptoms in caregivers and to measure the burden of care caregivers feel. Such research can improve early detection of depression in caregivers and help allocate necessary support. However, previous examinations of the reliability and validity of these two scales were conducted on primary caregivers of older people who require general nursing care, and 61.3% of the participants in the J-ZBI_8 verification had relatively mild care requirements (up to Care Level 2); thus, family caregivers who care for people with more urgent requirements, such as patients at the end of their lives, were not targeted.

Few studies have measured the care burden experienced by family caregivers who provide end-of-life care at home. One study focused on the effect caregivers' personalities have on perceived care burden [23], while another study explored the gap between patients in the terminal stage of dementia and their family caregivers in regard to their hope for treatment [24]. Further, Naoki et al. [15] used J-ZBI to study the care burden felt by family caregivers of cancer patients who were receiving palliative care at home (n = 23); however, only nine family caregivers eventually provided care at the end of the patients' lives, suggesting that the results cannot be adequately generalized to other family caregivers.

Tsai et al.'s [24] study disclosed that the preferences for end-of-life care differed between patients with dementia and their family caregivers, and Ishii et al.'s [6] study identified the difficulties facing family caregivers in providing end-of-life care for the patients with late stage of cancer at home. There are typical difficulties such as that of treatment in emergencies and spiritual pains, in family caregivers' experiences for the patients at the end-of-life phase. We hypothesized that family caregivers who provide end-of-life care at home experience a different situation than other caregivers. Further, the factor structure of ZBI is unclear and, while many researchers have attempted to verify it, differing models have been returned. We felt that it was necessary to confirm whether the 22-item single-factor J-ZBI and J-ZBI_8 (which has two factors and eight items) are appropriate scales for measuring the burden of family caregivers providing end-of-life care.

Consequently, the aim of this study was to examine the burden experienced by family caregivers who provide end-of-life care at home by using J-ZBI, and also to explore the J-ZBI structure.

## 2. Materials and Methods

### 2.1. Participants

To perform this study, we recruited a sample of family caregivers who were providing end-of-life care at home and who received home-care nursing services. These individuals were identified with the assistance of visiting nurses. Consequently, between October 2016 and March 2017, anonymous self-administered questionnaires were sent via mail to 389 visiting nurses, who in turn distributed them to 248 principal caregivers. Participants were informed of the aims of the investigation, and their written consent was obtained. The study protocol was approved by the Ethical Review Board of Yamanashi Prefecture University.

### 2.2. Questionnaire

We used J-ZBI to measure the care burden of the participating family caregivers. The survey included questions on basic demographics and caregiving topics (age, gender, number of secondary caregivers, and length of time using the service).

J-ZBI is composed of one factor and 22 items, each of which are scored using a scale ranging from 0 to 4; higher scores indicate a higher care-burden level.

In addition, J-ZBI_8 was also examined. As mentioned above, it is an abbreviated form of J-ZBI, featuring two factors (personal strain and role strain) and eight items.

We obtained permission to use J-ZBI for our study from Doctor Yumiko Arai on September 14, 2016.

### 2.3. Statistical Analysis

Item analysis was conducted using SPSS ver. 21.0 software package for Windows (SPSS, Chicago, IL, USA); factor extraction with Kaiser criteria was considered more appropriate to grasp multiple factors than Scree test [25–27]. Internal consistency and convergent validity was calculated. Additionally, Amos ver. 21.0 software package for Windows (AMOS, Chicago, IL, USA) was used to determine the compatibility of the models.

## 3. Findings

### 3.1. Questionnaire Response Rate

All 248 caregivers responded to the questionnaires. Of these respondents, 247 consented to participate in the study and were accepted as participants for analysis (effective response rate: 99.6%). The results are shown in Table 1.

### 3.2. Results of Item Analysis

For all items, values for skewness and kurtosis did not exceed ± 2; consequently these data were assumed to have normal distribution [28]. There were no missing values for any item in J-ZBI. Items 5, 11, 13, and 21 were found to have a ceiling or floor effect in terms of M ± 1SD. By removing these four items, all the rest of the items were regarded to have no significant distortion of normal distribution.

The theme of item 22 differed from those of the other items; therefore, these items were included in the subsequent statistical analysis. Characteristics of each item are shown in Table 2.

### 3.3. Results of Factor Extraction and Internal Consistency Calculation

The factor structure of the participants' care burden, obtained using J-ZBI, was identified using exploratory factor analysis (EFA). In the process of conducting the EFA, the Kaiser-Meyer-Olkin (KMO) measure of sampling adequacy and Bartlett's test of sphericity (X^2^) were also performed. Principal factor analysis was used for factor extraction, and promax rotation was also conducted. Further, a Kaiser criterion [29] was used to determine the number of factors involved. The KMO measure of sampling adequacy was determined to be 0.901, indicating that it was appropriate to use EFA to analyze the data; meanwhile, X^2^ was 1877.144 (df = 136) p < .001, indicating that this was an acceptable value. The attenuation situation of the four eigenvalues that were found to be higher than 1.0 were 6.821, 1.538, 1.202, and 1.132, and factor analysis showed that the number of factors was valid. The cumulative contribution rate was 62.89% for this four-factor model. Items with a factor loading of 0.4 or more were adopted as components of factors. Then, overload factors, including items that were related to “sacrificing life,” “personal strain,” “severe anxiety,” and “captivity,” were extracted in the EFA. Cronbach's *α* coefficients for the four factors after items with a low factor loading (less than 0.4) had been deleted were 0.864 (first factor: sacrificing life), 0.767 (second factor: personal strain), 0.670 (third factor: severe anxiety), and 0.703 (forth factor: captivity). These four factors, comprising 15 items, were grouped together and named J-ZBI_15. The results are shown in Table 3.

### 3.4. Validity of J-ZBI_15

To determine the convergent validity of J-ZBI_15, correlations between the four factors and item 22 were examined using Pearson's correlation analysis. Pearson's correlation analysis was considered appropriate because we used continuous variables and intended to conduct factor analysis on the assumption of normal distribution. Consequently, it was found that the correlation coefficient ranged from 0.330 to 0.766 (*p* < 0.01) among the four factors, indicating a weak–strong correlation.

**Figure 1:**
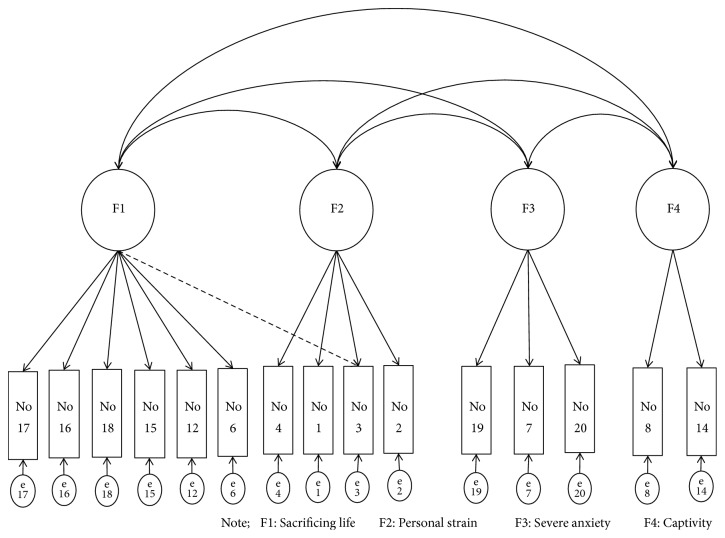
Four-factor structure: **χ**^2^/df ratio = 2.678 (222.307/83^*∗*^), CFI = 0.908, RMSEA = 0.083, AIC = 326.307.

Confirmatory factor analysis (CFA) was then conducted to explore the valid factor structure of the caregivers' care burden. The goodness-of-fit of the four-factor structure (J-ZBI_15), the original single-factor structure (J-ZBI), and the two-factor structure (J-ZBI_8) were confirmed, and the results were compared. Specifically, goodness of model fit for J-ZBI and J-ZBI_8 was confirmed using four indices (*χ*^2^/df ratio; comparative fit index (CFI); root mean square error of approximation (RMSEA); and Akaike information criterion (AIC)) and this was compared with the four-factor structure that was calculated using EFA. These results are shown in Table 4. For the four-factor structure model, *χ*^2^/df ratio = 2.678 (222.307/83, p < 0.001), CFI = 0.908, RMSEA = 0.083, and AIC = 326.307; the four-factor structure is shown in Figure 1. In contrast, the goodness-of-fit of the single-factor structure model (J-ZBI) was *χ*^2^/df ratio= 3.68 (770.71/209, p < 0.01), CFI = 0.78, RMSEA = 0.105, and AIC = 858.71, while for the two-factor structure model (J-ZBI_8) it was *χ*^2^/df ratio = 3.570 (67.846/19, p < 0.01), CFI = 0.932, EA = 0.102, and AIC = 117.84. These results are shown in Table 5.

## 4. Discussion

The aim of this study was to examine the burden experienced by family caregivers who provide end-of-life care at home by using J-ZBI, and also to explore the J-ZBI structure. The results showed the four-factor structure with 15 items (J-ZBI 15). This structure was different from the conventional structure as J-ZBI and J-ZBI 8 and had reliability and** v**alidity for use with family caregivers who provide end-of-life care at home. The following is a discussion of the results.

### 4.1. A Comparative Review of Previous Studies

In 2014, Cheng, Kwok, and Lam [30] performed a factor analysis on the ZBI, excluding item 22, and identified a four-factor model; F1: personal strain (nine items), F2: captivity (four items), F3: self-criticism (three items), and F4: loss of control (two items). In the present study, a four-factor model was also identified, with two factors, personal strain and captivity, consistent with those of Cheng et al.; however, the structure of the items was different. For example, Cheng et al.'s study included four questions concerning captivity: (1) “Do you feel that you don't have as much privacy as you would like because of your relative?” (2) “Do you feel that your social life has suffered because you are caring for your relative?” (3) “Do you feel uncomfortable about having friends over because of your relative?” (4) “Do you feel that your relative expects you to take care of him/her, as if you were the only one he/she can depend on?” Of these*, o*nly the fourth question is present in the model produced through this research.

Further, in Cheng et al.'s [30] study, the question, “Do you feel your relative is dependent on you?” (Item No 8) was classified as personal strain (degree of negative emotion felt towards a situation in which care is required); however, in this research it was classified as captivity. In fact, we consider the two items (Item No 8,14) that were classified as captivity in this research to be accurate means of indicating care burden relating to restraint. In addition, six items (Item No 6,12,15,16,17,18) that, in this study, were classified as sacrificing life, were classified across three factors (F1: personal strain, F2: captivity, and F4: loss of control) in Cheng et al.'s study.

As described above, the factor structure of the present study's 15-item four-factor model is completely different from the 18-item four-factor model in Cheng et al.'s [30] study. This might be because the previous study targeted family caregivers of patients with Alzheimer's disease, who may experience a different burden than that of caregivers providing end-of-life care at home.

J-ZBI_8 comprises two factors and eight items; F1: personal strain and F2: role strain [20, 31]. Again, this is totally different from our findings. Of the five items (Items Nos 4, 5, 9, 18, and 19) allocated to personal strain in J-ZBI_8, only two (Items Nos 4, 9) are included in personal strain in this study, and two of the three items (Items Nos 6, 12) constituting role strain in J-ZBI_8 were classified as sacrificing life in this study.

The care burden relating to providing end-of-life care at home is different from that of caregivers of individuals with other diseases such as Alzheimer's, for whom the 22-item ZBI is a characteristic model. In addition, we are also confident that the items in J-ZBI_8 are insufficient for accurately measuring the care burden of such end-of-life carers. In particular, in our model, captivity includes item 8 and item 14, which are not included in J-ZBI_8; these items are considered important for determining care burden, as they relate to caregivers' concern in regard to their care recipients and whether they remain preoccupied by this concern even when performing other tasks.

Thus, when measuring the care burden of individuals who provide end-of-life care at home in Japan, it is considered that using our 15-item model is most appropriate.

### 4.2. Examination of the Reliability and Validity of J-ZBI_15

In regard to reliability, Cronbach's *α* coefficients were 0.864, 0.767, 0.670, and 0.703 for the first (sacrificing life), second (personal strain), third (severe anxiety), and fourth (captivity) factors, respectively, as shown in Table 3. To confirm internal consistency, a Cronbach's *α* coefficient of > 0.6 is generally preferred [32]; thus, each factor is satisfactory.

In terms of validity, the correlation coefficient ranged from 0.330 to 0.766 (*p* < 0.01) between the four factors (J-ZBI_15) and item 22, indicating a weak–strong correlation. Therefore, it was considered that the four factors of J-ZBI_15 are capable of measuring caregivers' burden. Then, the four-factor structure (J-ZBI_15), the original single-factor structure (J-ZBI), and the two-factor structure (J-ZBI_8) were analyzed using CFA. The CFI and AIC scores for J-ZBI_15 were superior to those of J-ZBI_8, and the other indices (*χ*^2^/df ratio, RMSEA) also scored higher for J-ZBI_15. J-ZBI_8 best satisfied the general standard (*χ*^2^/df ratio < 3, CFI > 0.90, RMSEA < 0.08) [33]. Considering this, it can be determined that the four-factor structure with 15 items is* comprehensively* the most valid factor structure in this study.

*Limitations*. Some limitations exist in this study. First, the number of participants was small. There were 247 family caregivers who consented to participate, although we requested 389 family caregivers through visiting nurses. Second, J-ZBI is a self-administered tool, and so we had to check the answer after the description, because most of primary caregivers were old persons. Third, the difference in level of patients who were cared for by family caregivers affected our results. Although we measured care burden of primary family caregivers of those receiving end-of-life care at home, the stage of end-of-life should be assessed in future studies. Fourth, it is necessary to examine the difference in the care burden by participants' attributes: sexuality, age living together or separately, health condition, and financial conditions. Fifth, we did not assess the caregivers' emotional state and their ability to care. Prospective studies are required to know the association between care burden and care ability which includes the emotional state of family caregivers nationally and internationally. Identifying the association between care burden and care ability will give us many indications to improve end-of-life care at home by family caregivers. In addition, we believe that our findings are valuable, because they studied participants' information about caring for the patients currently, rather than after the patients' death; thus, we did not have to consider the grief of family caregivers.

More than half the Japanese people wish to die at home, but the rate of home deaths is 12.5% of the population, and that of those dying at hospital is 78.5% (Cabinet Office, 2013). To realize their wish of dying at home, home visit nurses need to improve their skills regarding end-of-life care. If home visit nurses could assess end-of-life care burden of family caregivers at home accurately by using the 15-item four-factor model, their care will improve, and they can intervene and provide more appropriate support to family caregivers by clarifying association with care ability. It is suggested to support the wishes of patients to die at home and increase end-of-life total care at home.

Thus, the J-ZBI 15 was standardized based on burden of family caregivers who provide end-of-life care at home. Therefore, when measuring burden of caregivers with other characteristics, factor structure of the J-ZBI should be examined again.

## 5. Conclusions

As a result of performing a factor analysis of the burden experienced by family caregivers who provide end-of-life care at home, a 15-item four-factor J-ZBI model was found to be most suitable for measuring this burden, with J-ZBI and J-ZBI_8 determined to be insufficient in this regard. Thus, this 15-item four-factor model should be used in the future to measure the burden experienced by such individuals.

We could facilitate the provision of more appropriate end-of-life total care for family caregivers by clarifying association between care burden and care ability.

## Figures and Tables

**Table 1:** Demographic variables of the primary caregivers.

Variables	Mean or Number	S.D. or %
Age	64.77	14.62
Gender		
Male	51	20.6%
Female	196	79.4%
Number of secondary caregivers	0.99	0.86
Duration receiving visiting nurses (months)	24.11	39.64

**Table 2:** The original version of the Zarit Caregiver Burden Interview [14].

No	Content of items	M	S.D.	Skewness	Kurtosis
1	Do you feel that your relative a**s**ks for more help than he/she needs?	1.35	1.10	.33	-.73
2	Do you feel that because of the time you spend with your relative that you don't have enough time for yourself?	1.91	1.09	.09	-.68
3	Do you feel stressed between caring for your relative and trying to meet other responsibilities for your family or work?	1.91	1.11	-.15	-.76
4	Do you feel embarrassed over your relative's behavior?	1.68	1.09	.11	-.74
5	Do you feel angry when you are around your relative?	0.98	1.01	.78	-.19
6	Do you feel that your relative currently affects your relationship with other family members or friends in a negative way?	1.40	1.16	.39	-.76
7	Are you afraid what the future holds for your relative?	1.96	1.24	.03	-.97
8	Do you feel your relative is dependent on you?	2.83	1.12	-.85	.43
9	Do you feel strained when you are around your relative?	1.28	1.12	.52	-.55
10	Do you feel your health has suffered because of your involvement with your relative?	1.17	1.09	.65	-.35
11	Do you feel that you don't have as much privacy as you would like because of your relative?	1.00	1.07	.87	-.06
12	Do you feel that your social life has suffered because you are caring for your relative?	1.49	1.21	.42	-.80
13	Do you feel uncomfortable about having friends over because of your relative?	1.06	1.21	.87	-.40
14	Do you feel that your relative seems to expect you to take care of him/her, as if you were the only one he/she could depend on?	2.04	1.46	-.10	-1.40
15	Do you feel that you don't have enough money to care for your relative, in addition to the rest of your expenses?	1.28	1.26	.54	-.91
16	Do you feel that you will be able to take care of your relative much longer?	1.44	1.22	.33	-1.07
17	Do you feel that you have lost control of your life since your relative's Illness?	1.63	1.11	.39	-.55
18	Do you wish you could just leave the care of your relative to someone else?	1.17	1.10	.63	-.42
19	Do you feel uncertain about what to do about your relative?	1.49	1.08	.34	-.67
20	Do you feel you should be doing more for your relative?	1.12	1.12	.76	-.23
21	Do you feel you could do a better job in caring for your relative?	0.94	1.03	.80	-.21
22	Overall, how burdened do you feel in caring for your relative?	2.03	1.07	.04	-.60

M: mean, S.D.: standard deviation.

**Table 3:** Four-factor structure in the present study.

No.	Content of items	F1	F2	F3	F4	Communality
F1: Sacrificing life (Cronbach's *α* coefficient= 0.864); 6 items						
17	Do you feel that you have lost control of your life since your relative's Illness?	**.954**	-.172	.009	.077	0.776
16	Do you feel that you will be able to take care of your relative much longer?	**.922**	-.028	.021	-.090	0.779
18	Do you wish you could just leave the care of your relative to someone else?	**.726**	.001	.145	-.236	0.563
15	Do you feel that you don't have enough money to care for your relative, in addition to the rest of your expenses?	**.604**	-.027	.082	-.112	0.358
12	Do you feel that your social life has suffered because you are caring for your relative?	**.511**	-.054	.073	.297	0.481
6	Do you feel that your relative currently affects your relationship with other family members or friends in a negative way?	**.483**	.135	.047	.147	0.473
F2: Personal strain (Cronbach's *α* coefficient= 0.767); 4 items						
4	Do you feel embarrassed over your relative's behavior?	-.082	**.752**	.157	-.120	0.556
1	Do you feel that your relative a**s**ks for more help than he/she needs?	-.229	**.642**	.165	.036	0.372
3	Do you feel stressed between caring for your relative and trying to meet other responsibilities for your family or work?	**.438**	**.573**	-.276	.017	0.654
2	Do you feel that because of the time you spend with your relative that you don't have enough time for yourself?	.241	**.523**	-.138	.137	0.502
9	Do you feel strained when you are around your relative?	.164	.350	.303	.042	0.511
F3: Severe anxiety (Cronbach's *α* coefficient=0.670); 3 items						
19	Do you feel uncertain about what to do about your relative?	.143	.100	**.647**	-.075	0.598
7	Are you afraid what the future holds for your relative?	.052	-.105	**.598**	.189	0.436
20	Do you feel you should be doing more for your relative?	.028	.123	**.414**	-.030	0.246
10	Do you feel your health has suffered because of your involvement with your relative?	.245	.210	.295	.065	0.446
F4: Captivity (Cronbach's *α* coefficient=0.703); 2 items						
8	Do you feel your relative is dependent on you?	-.056	-.014	-.026	**.780**	0.558
14	Do you feel that your relative seems to expect you to take care of him/her, as if you were the only one he/she could depend on?	-.099	.030	.107	**.685**	0.488
	Factor correlation					
	F1	1.000				
	F2	.685	1.000			
	F3	.562	.511	1.000		
	F4	.400	.399	.325	1.000	

Factor loadings with absolute values ≥0.40 are in boldface.

F: factor.

**Table 4:** Four-factor model (J-ZBI_15) and item 22 correlation.

	Sacrificing life	Personal strain	Severe anxiety	Captivity
Item 22 of J-ZBI	.766^*∗*^	.525^*∗*^	.494^*∗*^	.330^*∗*^

^*∗*^ *p* < 0.01.

**Table 5:** The goodness of fit of the models.

	*χ* ^2^/df ratio	CFI	RMSEA	AIC
Four-factor structure of J-ZBI_15	2.678 (222.307/83^*∗*^)	.908	.083	326.307
Original single-factor structure of J-ZBI	3.687 (770.719/209^*∗*^)	.782	.105	858.719

Two-factor structure of J-ZBI_8	3.570 (67.846/19)	.932	.102	117.846

^†^The four-factor structure was adopted after confirmatory factor analysis.

^*∗*^ *p *< 0.001

CFI: Comparative Fit Index

RMSEA: Root Mean Square Error of Approximation

AIC: Akaike Information Criterion.

## Abbreviations

J-ZBI: Japanese version of the Zarit Caregiver Burden Interview
EFA: Exploratory factor analysis
KMO: Kaiser-Meyer-Olkin
CFA: Confirmatory factor analysis
CFI: Comparative fit index
RMSEA: Root mean square error of approximation
AIC: Akaike's information criterion.
